# Supplementary material for: A multiplex analysis of phonological and orthographic networks
Source: PLoS One. 2022 Sep 15;17(9):e0274617. doi: 10.1371/journal.pone.0274617 (PMC9477335; doi:10.1371/journal.pone.0274617)
Supplement: S1 File — (PDF) [file pone.0274617.s001.pdf]

Supplementary Material

A multiplex analysis of phonological and orthographic networks

P. Lara-Martínez[1] B. Obregón-Quintana[1] F. Reyes-Manzano[2]
I. López-Rodríguez[3] L. Guzmán-Vargas[3]

[1]Facultad de Ciencias, Universidad Nacional Autónoma de México, Ciudad de México, México
[2] Tecnológico Nacional de México, Tecnológico de Estudios Superiores de Ixtapaluca, Km. 7 Carretera Ixtapaluca-Coatepec S/N San Juan, Ixtapaluca, Estado de México 56580, México
[3]Unidad Profesional Interdisciplinaria en Ingeniería y Tecnologías Avanzadas, Instituto Politécnico Nacional, Ciudad de México, México

We consider the probability degree-distribution functions for 72 (orthographic and phonological) networks. For each function, we performed fits to the data by considering the following distributions: Gumbel, Exponential, Loglogistic, Lognormal, Weibull and Power-law (see Table 1). In our procedure, we fit the data with maximum log-likelihood estimation (MLE).
To select the best fit we use the Akaike information criterion (AIC)[5] and the Bayesian information criterion (BIC) [1, 5]. These criteria are defined as:

$$AIC = -2log(\mathcal{L}(\hat{\theta}|y)) + 2K, \quad (1)$$

$$BIC = -2log(\mathcal{L}(\hat{\theta}|y)) + K\ln(n), \quad (2)$$

where K is the number of estimable parameters in the model, n is the number of data points and $\log(\mathcal{L}(\hat{\theta}|y))$ is the numerical value of the log-likelihood at its maximum point, with $\hat{\theta}$ the values that maximize the likelihood function and y are the data points.
After calculating the AIC for each of the fits, we rank them using the following increment ΔAIC_i^{exp} [1, 4], which is defined as:

$$\Delta AIC_i^{exp} = \exp((AIC_{min} - AIC_i)/2), \quad (3)$$

where AIC_{min} is the AIC for the top distribution in the ranking. The following condition [4], $\Delta AIC_i^{exp} < 0.01$, is applied to consider that the distribution with the AIC_{min}, is significantly better compared to the rest of the distributions [4]. A similar procedure was used by using the BIC index.
The results of the calculations described above are presented in Table 2. For each language, threshold value (ℓ) and layer, we compare all the fits in terms of their corresponding ΔAIC_i^{exp}, and ranking them to obtain the best model, i. e., how good it is compared to the other models. For orthographic distributions, we find that in 18 networks, they display a Weibull distribution, while for the other 18, the criteria indicate that the best fit correspond to Loglogistic (13 cases) and Lognormal (5 cases),

but the second candidate function corresponds to the Weibull (see Table 3). Regarding the phonological distributions, 19 cases correspond to a Weibull distribution, 11 to a Loglogistic and 6 to a Lognormal (see Table 3). We remark that Weibull, Loglogistic and Lognormal distributions are all heavy-tailed distributions, in which there are a few nodes (word-hubs) that concentrate most of the links. The specific values calculated for the parameters of the three most frequent distributions are in Table 4.
We obtained similar results when the models were compared by using the BIC index [1, 5]. Moreover, the application of the likelihood ratio test introduced by Clauset et al. [2, 3] has corroborated that in most cases, broad-tailed distributions (such as Weibull, Loglogistic or Lognormal) describe the data. Additional details of the tests performed (ACI, BIC criteria and ratio test) can be found in the CSV (statistical-Analysis_PDegree.csv) file that accompanies the online information on https://doi.org/10.6084/m9.figshare.14668593.

Distribution	pdf		
Exponential	$f_{(\lambda,\gamma)}(x)$	$=$	$\lambda e^{-\lambda(x-\gamma)}$
Gumbel	$f_{(\alpha,\lambda)}(x)$	$=$	$\frac{1}{\lambda}e^{z-e^{z}}$, where $z=\frac{x-\alpha}{\lambda}$
Loglogistic	$f_{(\alpha,\lambda,\gamma)}(x)$	$=$	$\frac{\left(\frac{\alpha}{\lambda}\right)\left(\frac{x-\gamma}{\lambda}\right)^{\alpha-1}}{\left(1+\left(\frac{x-\gamma}{\lambda}\right)^{\alpha}\right)^{2}}$
Lognormal	$f_{(\alpha,\lambda,\gamma)}(x)$	$=$	$\frac{1}{\lambda(x-\gamma)\sqrt{2\pi}}exp\left(-\frac{1}{2}\left(\frac{ln(x-\gamma)-\alpha}{\lambda}\right)^{2}\right)$
Power-Law	$f_{(\alpha)}(x)$	$=$	$Cx^{-\alpha}$, where C is the normalization factor.
Weibull	$f_{(\alpha,\lambda,\gamma)}(x)$	$=$	$\lambda\alpha\left(\lambda(x-\gamma)\right)^{\alpha-1}e^{-(\lambda(x-\gamma))^{\alpha}}$

Table 1: List of distribution functions considered in our study.

References

[1] D Anderson and K Burnham. Model selection and multi-model inference. *Second. NY: Springer-Verlag*, 63(2020):10, 2004.

[2] Aaron Clauset, Cosma Rohilla Shalizi, and Mark EJ Newman. Power-law distributions in empirical data. *SIAM review*, 51(4):661–703, 2009.

[3] Aaron Clauset, Maxwell Young, and Kristian Skrede Gleditsch. On the frequency of severe terrorist events. *Journal of Conflict Resolution*, 51(1):58–87, 2007.

[4] Ernesto Estrada and Grant J Ross. Centralities in simplicial complexes. applications to protein interaction networks. *Journal of theoretical biology*, 438:46–60, 2018.

[5] Sadanori Konishi and Genshiro Kitagawa. Information criteria and statistical modeling. 2008.

Language	Threshold ℓ	Orthographic	Phonological
Dutch	1	Loglogistic	Loglogistic
	2	Weibull	Weibull
	3	Loglogistic	Weibull
English	1	Weibull	Weibull
	2	Weibull	Weibull
	3	Weibull	Weibull
French	1	Weibull	Weibull
	2	Lognormal	Loglogistic
	3	Loglogistic	Weibull
German	1	Loglogistic*	Weibull
	2	Weibull	Weibull
	3	Loglogistic	Loglogistic
Hungarian	1	Loglogistic	Loglogistic
	2	Weibull	Weibull
	3	Loglogistic	Loglogistic*
Italian	1	Weibull	Loglogistic
	2	Lognormal	Lognormal
	3	Loglogistic	Loglogistic
Polish	1	Weibull	Weibull
	2	Lognormal	Lognormal
	3	Loglogistic	Loglogistic
Portuguese	1	Weibull	Weibull
	2	Lognormal	Lognormal
	3	Loglogistic	Loglogistic
Russian	1	Weibull	Loglogistic
	2	Weibull	Weibull
	3	Loglogistic	Lognormal
Spanish	1	Weibull	Weibull
	2	Lognormal	Lognormal
	3	Loglogistic	Loglogistic
Swedish	1	Weibull	Weibull
	2	Weibull	Weibull
	3	Weibull	Weibull
Ukrainian	1	Weibull	Weibull
	2	Weibull	Weibull
	3	Loglogistic	Lognormal

Table 2: Results for the 72 degree distributions in our study. The best fit was obtained in terms of the *AIC* and *BIC* criteria. The Loglogistic distributions marked with (*), represent the cases for which the AIC (BIC) criterion exhibits a weak differentiation with the second best distribution (Weibull).

Distribution	Orthographic	Phonological
Exponential	0	0
Gumbel	0	0
Loglogistic	13	11
Lognormal	5	6
Power-Law	0	0
Weibull	18	19

Table 3: For each distribution, we list the number of networks (either orthographic or phonological) for which AIC and BIC criteria were significant to decide between the distributions.

Language	Threshold	Orthographic						Phonological					
		Weibull		Lognormal		Loglogistic		Weibull		Lognormal		Loglogistic	
		$\hat{\alpha}$	$\hat{\lambda}$	$\hat{\alpha}$	$\hat{\lambda}$	$\hat{\alpha}$	$\hat{\lambda}$	$\hat{\alpha}$	$\hat{\lambda}$	$\hat{\alpha}$	$\hat{\lambda}$	$\hat{\alpha}$	$\hat{\lambda}$
	1	0.26	2.13	-3.17	5.06	1.30	1.46	0.25	2.21	-3.28	5.16	1.16	1.44
Dutch	2	0.35	0.11	0.32	4.57	0.43	3.39	0.32	0.15	-0.18	4.83	0.39	2.09
	3	0.41	0.01	2.77	3.80	0.56	27.25	0.39	0.02	2.14	4.09	0.52	16.73
	1	0.63	0.35	-2.38	5.02	0.32	0.17	0.28	0.52	-1.64	5.09	0.33	0.41
English	2	0.41	0.05	1.36	4.06	0.53	8.21	0.39	0.03	1.74	4.19	0.50	11.90
	3	0.49	0.01	4.21	2.28	0.68	73.25	0.47	0.00	4.42	2.45	0.63	94.44
	1	0.28	1.71	-2.80	4.88	0.33	0.10	0.39	0.20	-0.15	4.31	0.47	2.17
French	2	0.50	0.08	1.97	1.68	0.67	6.79	0.51	0.02	3.27	1.95	0.73	26.83
	3	0.57	0.01	3.87	1.81	0.84	48.57	0.57	0.00	5.26	2.04	0.79	210.19
	1	0.89	0.45	-2.84	4.89	1.90	2.09	0.58	0.46	-2.99	5.12	0.31	0.07
German	2	0.38	0.14	0.19	4.39	0.46	3.01	0.34	0.11	0.25	4.59	0.43	3.13
	3	0.42	0.02	2.41	3.83	0.56	19.98	0.39	0.02	2.44	3.97	0.53	20.71
	1	0.24	5.82	-4.23	4.97	1.81	1.37	0.24	5.88	-4.24	4.97	0.73	0.08
Hungarian	2	0.39	0.19	-0.13	4.35	0.46	2.22	0.37	0.22	-0.35	4.50	0.43	1.81
	3	0.46	0.03	2.73	2.23	0.63	16.62	0.44	0.03	2.03	3.74	0.59	13.99
	1	0.30	1.27	-2.45	4.83	0.34	0.17	0.30	1.15	-2.34	4.84	1.64	2.13
Italian	2	0.48	0.07	1.98	1.90	0.63	7.23	0.47	0.06	2.00	1.96	0.63	7.55
	3	0.54	0.01	3.95	1.93	0.78	53.15	0.53	0.01	3.94	1.97	0.77	52.97
	1	0.29	1.63	-2.70	4.81	0.34	0.12	0.28	2.06	-3.01	4.88	0.33	0.08
Polish	2	0.49	0.10	1.73	1.68	0.63	5.26	0.48	0.10	1.68	1.73	0.62	5.06
	3	0.55	0.01	3.55	1.84	0.80	35.25	0.53	0.01	3.47	1.87	0.78	32.22
	1	0.29	1.66	-2.75	4.85	0.33	0.11	0.29	1.36	-2.54	4.85	0.34	0.15
Portuguese	2	0.50	0.07	1.97	1.77	0.65	6.95	0.48	0.07	2.02	1.85	0.64	7.46
	3	0.56	0.01	3.92	1.86	0.82	51.37	0.55	0.01	3.97	1.88	0.81	53.83
	1	0.25	4.81	-3.96	4.90	0.32	0.02	0.24	6.20	-4.25	4.91	2.79	1.60
Russian	2	0.44	0.17	0.21	4.04	0.53	2.99	0.40	0.22	-0.21	4.24	0.48	2.06
	3	0.51	0.02	3.11	1.92	0.74	22.66	0.48	0.03	2.65	2.02	0.67	14.53
	1	0.27	2.48	-3.23	4.91	0.32	0.06	0.29	1.39	-2.57	4.87	0.33	0.14
Spanish	2	0.48	0.09	1.76	1.84	0.61	5.71	0.49	0.06	2.10	1.85	0.65	8.12
	3	0.56	0.01	3.77	1.84	0.81	43.90	0.56	0.01	4.10	1.89	0.81	61.36
	1	0.27	1.78	-2.90	4.96	0.32	0.09	0.26	1.91	-3.05	5.06	0.31	0.07
Swedish	2	0.38	0.10	0.54	4.40	0.46	4.20	0.35	0.11	0.20	4.70	0.41	3.13
	3	0.42	0.01	2.86	3.84	0.55	30.87	0.40	0.02	2.50	4.07	0.52	23.36
	1	0.25	3.84	-3.73	4.93	0.32	0.03	0.25	4.97	-4.02	4.93	0.31	0.02
Ukrainian	2	0.46	0.13	0.54	3.88	0.57	3.89	0.43	0.16	0.21	4.04	0.53	2.97
	3	0.52	0.02	3.32	1.93	0.75	27.93	0.49	0.02	2.98	1.98	0.71	19.90

Table 4: Estimated values of the parameters for the degree distributions. We show the cases of Weibull, Lognormal and Loglogistic, which were the most frequent funtions in our analysis.
