# Supplementary material for: A multiplex analysis of phonological and orthographic networks
Source: PLoS One. 2022 Sep 15;17(9):e0274617. doi: 10.1371/journal.pone.0274617 (PMC9477335; doi:10.1371/journal.pone.0274617)
Supplement: Attachment — Submitted filename: ResponsesPlosOne2022.pdf [file pone.0274617.s002.pdf]

Response to Referees

May 2022

Response to Reviewers

Reviewer 1

1. *The authors analyze the topological properties of phonological and orthographic networks of 12 languages by adopting a multiplex formalism. They perform a comprehensive analysis of these graphs and uncover interesting patterns of language use.*
   *Strength * The report is well-prepared, and the analysis is comprehensive.*
   *Weakness * The figures and captions are out of place. The authors should consider redesigning all the figures to fit within the text, even if it involves splitting the figures into separate subfigures.*
   I recommend the authors fix the figures (see above) as a minor revision to make this fit for publication.
   Response: Thank for your comments and suggestions. We have redesigned all the figures as suggested.

Reviewer 2

1. *My main criticism of the article is that it seems that a series of very interesting analyzes have been carried out but without a clear objective. For example, in the abstract, it is said: "We report that from the analysis of topological properties of networks, there are different levels of local and global similarity when comparing..." This result somehow would be expected. The interesting question would be univocally framing those results with other linguistic research, phylogenetic approaches, information theory or sociology hypothesis of language. I believe that the article has great potential, but it should make it more straightforward about the hypotheses, objectives, and substantial contributions beyond an exciting series of analyses.*
   Response: We welcome comments on the submitted version. These observations have been very constructive and are greatly appreciated. We have revised and added corrections according to the opinions expressed. Particularly, we have clarified the

scope of the study in terms of objectives, hypotheses and contributions.

2. *Additionally, I would like to propose the following comments: Introduction.*
   Some sentences are vague, e.g. "The complexity of natural language has been studied from different perspectives of scientific research". This kind of sentence can be more concrete and explicative, including references to previous works.
   Response: We have re-written these sentences in a more detailed way and added citations.

3. *Line 9-13: This should be referenced.*
   Response: We have added the corresponding citations.

4. *Line 13.15: However, it was later fitted to a Weibull distribution.*
   Response: Thank you for the annotation. In previous reports, the case of phonological networks constructed from similarities in sounds led to truncated power law type degree distributions (we have added ref. ([20])). For our part, the similarity is based entirely on the Damerau-Levenhstein distance, which, although relatively easier to calculate, tends to identify greater similarity between words, hence there is a difference between the functions that best approximate the distributions.

5. *Related to ref 5 and the relationship between ortographic and phonology I would suggest to consider the reference: Torre, I. G., Luque, B., Lacasa, L., Kello, C. T., Hernández-Fernández, A. (2019). On the physical origin of linguistic laws and lognormality in speech. Royal Society open science, 6(8), 191023; where the authors have addressed this kind of statistical regularities of language.*
   Response: Thank you very much for bringing us closer to the work of Torre et. al, which is very interesting in the context of the universality and origin of linguistic laws. We have added the corresponding citation.

6. *The relationship between orthographic and phonology networks could be of great interest to computer science, where automatic speech recognition systems may fail to transcribe into written text. This somehow could be related to the transfer learning between languages: e.g. a neural network that can recognize a language could work well in another language depending on the similarity of the multiplex network.*
   Response: We appreciate this interesting suggestion on the usefulness of phonetic-orthographic networks. We have added a few sentences related to this as motivation in the Introduction (see lines 50-55)

7. *Limitation of ortographic translation of Damerau-Levenshtein. The orthographic transcription is, in some way, a discrete representation of the spoken word, but the orality is a continuum spectrum. In linguistics, the vowels are represented in the IPA vowel trapezium, some of them physically closer to others. The Damerau-Levenshtein distance does not recover this fact.*
Response: We agree that the similarity based on the Damerau-Levenhstein distance has limitations that impact especially in phonetic network, where this affinity is overestimated because the discretization of sounds leads to loss of structure. We have added a few sentences pointing out these limitations.

8. *Material and methods, and Results*
Degree distributions are fitted with a Weibull-type function. This hypothesis should be supported by literature and compared to another type of distribution function, e.g. power-law or exponential.
Response: We have extended our description and justification of fitting with a Weibull function (see lines xx-xx)

9. *The fitting to a probability distribution function, particularly a power law, has been a hot topic in recent years, so it should be rigorously addressed. The article should include the methodology used, the estimators and the goodness of fit. Some useful references: • Clauset, A., Shalizi, C. R., Newman, M. E. (2009). Power-law distributions in empirical data. SIAM review, 51(4), 661-703. • Navas-Portella, V., González, Á., Serra, I., Vives, E., Corral, Á. (2019). Universality of power-law exponents by means of maximum-likelihood estimation. Physical Review E, 100(6), 062106.*
Response: We appreciate the suggestions for a more rigorous justification of the selection of the best fit to the distributions. We have added the corresponding citations and included a description of the tests used.

10. *Line 144. The Pearson correlation test hypothesis should be revisited. It usually requires a normal distribution of the values, which seems to be not happening, at least for the degree.*
Response: Thank you for the observation of the distribution requirement. We have reviewed the presence of correlations in terms of Spearman rank correlation coefficient. Unlike the case of Pearson's correlation, it does not require the distributions to be normal. The results are relatively similar.

11. *Material and methods should be extended to detail the mathematical approaches to fit the distribution and goodness of fit analysis.*
Response: We have added details of our procedure to fit the distributions and the goodness of fit analysis (see changes in lines 309-318)

12. *Discussion and Conclusions.*
There is no apparent difference between the message written in the discussion and the one written in the conclusion section. I would suggest being more specific or merging both sections.
Response: We have reorganized these parts and merged both Sections

13. *Reproducibility of the article.*
While the data has been shared, I would recommend to also sharing the scripts used during calculations.

Response: The scripts of the most representative calculations are now shared in the FigShare link.
