# Supplementary material for: A multiplex analysis of phonological and orthographic networks
Source: PLoS One. 2022 Sep 15;17(9):e0274617. doi: 10.1371/journal.pone.0274617 (PMC9477335; doi:10.1371/journal.pone.0274617)
Supplement: Attachment — Submitted filename: ResponsesRound3PlosOne2022.pdf [file pone.0274617.s003.pdf]

Response to Referees

May 2022

Response to Reviewers

Reviewer 1

1. The revised manuscript addresses the concerns raised by R2. However, the issues raised by R1 regarding the misplaced figures still exists. This could be an artifact of TeX-diff, in which case, the authors should ignore the above concerns.
   Response: We have corrected the Figures.

Reviewer 2

1. *Although, in general, the authors have reviewed the comments and worked on the article, I have doubts about one of the points: the adjustments of the network degree distribution. As was stated in the previous version, there has been a lot of research and discussion on this topic during the last decade. Therefore, the fit of a degree distribution by the least-squares method is not recommended at all (A Clauset · 2007, 2009, Anna Deluca Álvaro Corral, 2015; among others). One option is applying the maximum likelihood method to adjust the parameters and detect the lower cut-off points, but other statistical methods are available. Furthermore, different candidate distributions should be considered when the slopes are not very steep (as seems to be the case), typically Weibull, lognormal, power law, gamma or exponential. MLE numerical results should be provided with the values of loglikelihood, BIC or AIC. It should be considered that some of these distributions have a different number of variables. The best candidate distribution should be selected with loglikelihood, AIC or BIC. Finally, the goodness of fit should also be provided. The results of those fitting should be added to the main document or supplementary material, and ideally, the programming scripts should also be provided.*

   Response: We appreciate the suggestion to explore in more detail the problem of fitting empirical degree distributions. We have adopted this strategy and several functions have been considered. Our approach has been based, as suggested, on the AIC and BIC indices, to identify the best fit. In addition, we

have used the reason methodology proposed by A. Clauset et al. to confirm that the candidate distribution is better compared to the other distributions (see lines 134-157 of the main article and the Supplementary Material in FigShare (`https://doi.org/10.6084/m9.figshare.14668593` **). Additional details, such as the results of the fittings, test values and programming scripts performed are also shown in the material available online.**
